# Risk assessment in patients with an acute ST-elevation myocardial infarction

**DOI:** 10.2217/cer-2016-0017

**Published:** 2016-09-01

**Authors:** Nadeem Ahmed, Jaclyn Carberry, Vannesa Teng, David Carrick, Colin Berry

**Affiliations:** 1 BHF Glasgow Cardiovascular Research Centre, Institute of Cardiovascular & Medical Sciences, University of Glasgow, Glasgow, UK; 2 Golden Jubilee National Hospital, Clydebank, UK

**Keywords:** cardiac MRI, cardiology/cardiovascular, coronary angiography, echocardiography, evidence-based medicine, invasive hemodynamics, plasma biomarkers, risk assessment, risk scoring, STEMI

## Abstract

ST-elevation myocardial infarction (STEMI) is one of the leading causes of mortality and morbidity worldwide. While the survival after acute STEMI has considerably improved, mortality rate still remains high, especially in high-risk patients. Survival after acute STEMI is influenced by clinical characteristics such as age as well as the presence of comorbidities. However, during emergency care increasing access to tools such as the electrocardiogram, chest x-ray and echocardiography can provide additional information helping to further risk stratify patients. In the invasive setting, this can also include coronary angiography, invasive hemodynamic recordings and angiographic assessments of coronary flow and myocardial perfusion. We outline the common investigations used in STEMI and their role in risk assessment of patients with an acute STEMI.

ST-elevation myocardial infarction (STEMI) is one of the leading causes of mortality and morbidity worldwide. However, survival after acute STEMI has considerably improved due to increasing symptom recognition, accurate diagnosis and effective timely reperfusion [1]. Further reasons for reduction in STEMI mortality can be explained by greater use of percutaneous coronary intervention (PCI), antithrombotic therapy and secondary cardiovascular prevention strategies [1–3]. The incidence of STEMI has seen a general decline in recent years [4]. Despite this, mortality remains substantial with approximately 12% of patient’s dead within 6 months and with higher mortality rates noted in high-risk patients [5].

Survival after an acute STEMI is influenced by characteristics such as age, comorbidities (diabetes mellitus [DM], hypertension, previous myocardial infarction [MI] and renal failure), multivessel disease (MVD), left ventricular (LV) ejection fraction (EF) and timely revascularization (thrombolysis and/or PCI).

## STEMI definition

STEMI is a part of a spectrum of acute coronary syndromes (ACS), which also include non-STEMI (NSTEMI) and unstable angina (UA). The European Society of Cardiology (ESC) guidelines define STEMI as a rise and/or fall in cardiac biomarkers with at least one of the following symptoms of ischemia, new ST changes or new left bundle branch block, appearance of pathological Q waves, imaging evidence of loss of viable myocardium and finally the confirmation of intracoronary thrombus by coronary angiography [1].

## Initial presentation

### Clinical characteristics

**Figure 1.**
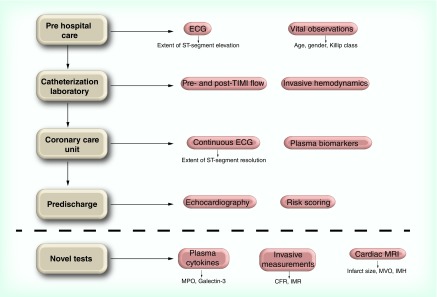
**Routine investigations used in ST-elevation myocardial infarction patients in clinical practice.** CFR: Coronary flow reserve; IMH: Intramural hematoma; IMR: Index of microcirculatory resistance; MPO: Myeloperoxidase; MVO: Microvascular obstruction; TIMI: Thrombolysis in myocardial infarction.

Early risk assessment at the time of the first medical contact is important. Clinicians should consider the age of the patient, history of comorbidity and examination findings including vital signs and presence of heart failure (HF) (Figure 1).

### Gender, age, comorbidities & Killip classification

Increasing age and presence of comorbidities have been recognized as critical factors in predicting clinical outcome in patients with STEMI. Comorbidities leading to higher incidence of early mortality include existing coronary heart disease, DM, renal disease, peripheral vascular disease and HF. A large cohort study (n = 28,421) in Korea conducted by Park *et al.* found that independent predictors of early mortality in STEMI patients included old age above 60 years old, Killip class, female gender, LV dysfunction and renal failure [6]. Another study by Pederson *et al.* revealed that DM, in addition to age and HF, were found to be independent predictors of early mortality in STEMI patients [7]. The prognostic value of gender has been a controversial topic in STEMI patients. Some studies have shown female gender to be an independent predictor for mortality in STEMI patients. For example, a large observational study in Paris by Benamer *et al.*, which included 16,760 STEMI patients, demonstrated that female gender is independently associated with higher inpatient mortality rate in a multivariate analysis [8]. Conversely, a multihospital study by Kyto *et al.* on 31,689 STEMI patients revealed that the differences in in-hospital mortality between genders were caused by differences in age and comorbidities and not due to gender itself [9].

The Killip classification, which categorizes patients based on the severity of HF (Killip class I – no evidence of HF, through to Killip class IV – cardiogenic shock), has been a valuable tool for early risk stratification in STEMI. The Global Registry of Acute Coronary Events (GRACE) investigators evaluated 16,166 acute MI patients and demonstrated that HF on admission was associated with four-times greater risk of in-hospital mortality, across all ACS subsets [10].

### Cardiogenic shock & Glasgow Coma Scale

Cardiogenic shock (hypotension, inotrope, vasopressor or intra-aortic balloon pump use or end-organ hypoperfusion) occurs in approximately 5% of STEMI patients and is associated with an in-hospital mortality rate of 38% [11]. Examination findings, such as heart rate and blood pressure on admission have predictive value on the short-term clinical outcomes post PCI in STEMI. They have been used in several risk assessment tools including GRACE and thrombus in myocardial infarction (TIMI) score. A low systolic blood pressure (<100 mmHg) and high heart rate (>100 bpm) are associated with poor short-term clinical outcomes. An observational study on 2310 STEMI patients treated with primary PCI showed that increased heart rate on admission was an independent prognostic factor for in-hospital and long-term mortality [12,13].

The finding of a new murmur is an indicator of a structural complication of STEMI, which may lead to acute HF. STEMI with inferior ischemia can lead to papillary muscle dysfunction causing mitral regurgitation, which results in new pansystolic murmur. It rarely occurs with anterior or lateral ischemia due to the dual blood supply to the anterolateral papillary muscle. Ischemic mitral regurgitation is a common complication of STEMI and has been known to be an independent strong predictor of long-term mortality [13]. However, no studies have proven that mitral regurgitation in STEMI is associated with adverse short-term clinical outcomes. Acute septal rupture is a life threatening complication that can present with cardiogenic shock, biventricular failure and new pansystolic murmur, usually within the first week post MI.

A prospective study by Lettieri *et al.*, which included 2617 patients with STEMI treated with emergency PCI in 2005, found that a Glasgow Coma Scale (GCS) score of 3 was an independent predictor of in-hospital mortality [14]. This result was consistent with a China-based study by Pan *et al.*, which demonstrated that GCS ≤7 and cardiogenic shock, on admission were independent predictors of early mortality, in patients with out-of-hospital cardiac arrest [15]. Both studies also showed that long-term mortality risks in these patients were similar to those without prehospital cardiac arrest. However, it is possible that the consequential adverse short-term outcomes may be confounded by cardiac arrest events. There is still uncertainty in the role of the GCS score on admission for risk stratification.

## In-hospital course

### Coronary angiography

#### Extent of coronary artery disease

Around 40–50% of patients presenting with STEMI have MVD and these patients have been consistently shown to have worse cardiac risk profiles than patients with single-vessel disease [16]. Many early studies (before the stenting era) found the presence of MVD to be a predictor of 30-day or in-hospital mortality, independent of other cardiovascular risk factors [17]. However, since the introduction of stenting, the association between MVD and short-term outcome is less convincing. Sorajja *et al.* found a higher frequency of recurrent ischemia in MVD patients at 30 days, but a nonsignificant trend toward death and major adverse cardiac events (MACE) at 30 days [16]. Similarly, Dziewierz *et al.* saw that patients with MVD had an increased risk of ischemic events, bleeding at 30 days and an association with 30-day mortality that was dependent on cardiogenic shock [18].

The prognostic significance of MVD in assessing acute risk may be dependent on the presence of a chronic total occlusion (CTO) [19]. In a study of 3277 STEMI patients conducted in Amsterdam, the presence of a CTO in a noninfarct related artery was a strong predictor of 30-day mortality (hazard ratio [HR]: 3.6 [95% CI: 2.6–4.7]; p < 0.01), while MVD without a CTO was a comparably weaker predictor (HR: 1.1 [95% CI: 0.8–1.5]; p = 0.01) [19]. These results were further replicated in the HORIZON-AMI patient cohort [20]. Current evidence suggests that the early prognostic value of MVD in assessing risk may be driven by the presence of CTO and cardiogenic shock.

Similarly the presence of multiple complex plaques, identified on angiography, and defined as more than one lesion with two or more defining features of a complex plaque, are associated with MACE and major bleeding at 30 days [21]. Additionally, these patients are more likely to require urgent bypass surgery [21]. Multivariable analysis confirms that the presence of multiple complex plaques is an independent predictor of 3-year MACE [21].

#### Hemodynamics: LV end-diastolic pressure, central venous pressure & right heart, pulmonary capillary wedge pressure

While there have been studies to suggest that hemodynamic measures can predict 30-day mortality and infarct size (IS), the evidence of the prognostic value of hemodynamic measures in STEMI patients is limited [22], partly because right heart catheterization is no longer performed due to associated risks. LV end-diastolic pressure is frequently measured at PCI, however, and has been shown to be an independent predictor of in-hospital death, cardiogenic shock and HF [23]. Furthermore, LV end-diastolic pressure >18 mmHg predicts death and re-infarction 30 days post STEMI [24,25].

#### Left ventriculography

LV involves the injection of radiographic dye via a multisidehole pigtail catheter inserted retrogradely through the aortic valve into the LV to assess LV function and volume [26]. The procedure provides information on wall motion, LV thrombus (LVT), mitral regurgitation, aneurysms and ventricular septal defects (VSDs) as well as aids the diagnosis of Takotsubo cardiomyopathy, an STEMI mimic [26]. However, the procedure poses significant risks to the patients, including longer radiation exposure, increased contrast use, embolization or myocardial rupture [26]. Furthermore, there are alternate noninvasive methods for measuring LV volumes and function, including cardiac MRI and echocardiography [26]. Moreover, left ventriculography has shown variable performance when compared with echocardiography in patients with MI [27]. While left ventriculography is a low-cost procedure and may be of use when LV function is unknown, its routine use as a prognostic indicator in STEMI is not indicated.

## Angiographic assessments of coronary flow & myocardial perfusion

### TIMI flow grade

The TIMI flow grade (TFG) is an angiographic measure of flow in an epicardial artery and is graded 0, 1, 2 or 3 (0 – no flow; 1 – minimal flow past obstruction; 2 – slow but complete filling and slow clearance; 3 – normal flow and clearance) (Table 1). Corrected TIMI frame count is a more objective and quantitative index of coronary flow, which is calculated by counting the number of cineframes needed for dye to reach standardized distal landmarks [28].

**Table 1.** **Thrombolysis in myocardial infarction blush grade.**

**TIMI blush grade**	**Definition**
0	No myocardial blush

1	Minimal blush and very slow clearing (e.g., present at the beginning of next cine)

2	Good blush with slow clearing of myocardial contrast (present at the end of cine but gone at the beginning of next)

3	Good blush and normal clearing (i.e., gone by the end of cine)

TIMI: Thrombolysis in myocardial infarction.

TFG before intervention has been studied in detail in the context of pharmacological and mechanical interventions to achieve patency in the culprit artery. Notably, STEMI patients with TFG 2–3 in the culprit artery after thrombolysis and before mechanical intervention, were seen to have higher rates of TFG 3 post procedure, smaller ISs, better predischarge LVEFs, shorter hospital stays and lower risk of cardiogenic shock [25,29]. In addition, patients presenting with culprit TFG 2–3 have been shown to have better 30-day survival compared with those with TFG 0–1 [25,29].

TFG post procedure has been consistently shown to be associated with in-hospital mortality in STEMI patients, including in specific subpopulations of cardiogenic shock patients and elderly patients (≥75 years) [30,31]. Studies also report higher rates of 30-day mortality and 30-day MACE in patients with impaired TFG after intervention [32]. Post procedural TFG is also included in both the CADILLAC score and Angiographic Perfusion Score (TFG + TIMI myocardial perfusion grade [TMPG]), which both predict 30-day mortality [33]. There is a wealth of evidence to suggest that the TFG, both before and after interventional treatment, is an indicator of MI severity and short-term outcomes.

### Myocardial perfusion & the no-reflow phenomenon

Angiographic successful reperfusion in acute MI has been defined as TIMI 3 flow. However, TIMI 3 flow does not always result in effective myocardial reperfusion. Myocardial blush grade is an angiographic measure of myocardial perfusion and is therefore used to assess perfusion in the capillary bed at the tissue level. The TIMI blush grade is an ordinal score for contrast washout at the end of the angiogram, and the TIMI blush grade is also predictive of prognosis.

Gibson *et al.*, first assessed the associations with TMPG in a large cohort of patients (n = 762) and reported that patients with TMPG 0 had a higher mortality rate at 30 days compared with those with TMPG 1, 2 or 3 [34]. Importantly, the association held true when restricted to patients with TFG 3, anterior MI and patient characteristics such as age and gender [34]. Later studies associated a low TMPG post revascularization with the incidence of an ischemic event within 48 h [35], and ventricular arrhythmia within 30 days of thrombolysis [36]. In addition, TMPG has been shown to be in concordance with the extent of ST-segment resolution after treatment for STEMI. While associations with both absolute and relative ST-segment changes were significant, they were weak suggesting other factors may be relevant.

### Index of microcirculatory resistance

Coronary guidewire-based sensor technologies have emerged as new diagnostic tools for the invasive assessment of coronary artery disease in the setting of ACS. The index of microvascular resistance (IMR) is a simple coronary guidewire-based method for assessing coronary microvascular function and is a direct measure of microvascular resistance. IMR measured directly after primary PCI predicts IS [37] and functional recovery [38]. In a landmark recent study, 253 acute STEMI patients with IMR >40 had a higher rate of the primary end point of death or rehospitalization for HF at 1 year than patients with an IMR ≤40 (17.1 vs 6.6%; p = 0.027) [39]. IMR is promising for early risk stratification in acute reperfused STEMI survivors, since it is readily available in the catheter laboratory, quantitative and reproducible. On the other hand, evidence is lacking that treatment decisions linked to IMR result in clinical benefit, and in this regard, more research is warranted.

### Coronary flow reserve

Coronary flow reserve (CFR) is another coronary guidewire-based method, which reflects epicardial and microvascular vasodilator capacity. CFR is more amenable to hemodynamic changes and is less reproducible than IMR [40]. Nevertheless, Cuculi *et al.*, showed that change in CFR in the first day after primary PCI was associated with 6-month LVEF and myocardial salvage (MS) [41]. Van de Hoef *et al.* found that in 157 STEMI survivors, a CFR <2.0 was associated with MACE over a 10-year period [42]. Prior studies of IMR and CFR are limited by sample size, short follow-up and differences in cut-offs, supporting the case for further research.

## Post reperfusion (coronary care unit)

### ECG

ECG monitoring should be continuous throughout initial reperfusion period, and a 12-lead ECG should be acquired 60–90 min post reperfusion to asses for successful myocardial perfusion. This should show resolution of ST elevation and hyperacute T-waves. Indeed, the extent of early resolution of ST segment has been linked to IS and EF [43]. On the contrary persistent ST-elevation after reperfusion may be associated with need for further interventions due to more extensive myocardial damage and a higher mortality rate [43]. Furthermore, ECG reperfusion provides a real-time physiological marker of cellular perfusion and is a significant predictor of LV contractility recovery. Ideally, there needs to be continuous ECG recording to monitor segment resolution. Early reperfusion of the terminal portion of T-waves after the initiation of reperfusion therapy is another indicator of successful reperfusion.

### Plasma biomarkers

#### Cardiac troponin: standard assay (µg) & high-sensitivity assay (ng/l)

The sensitivity and specificity of different commercially troponin assays vary considerably relating to lack of standardization, different use of monoclonal antibodies and the presence of modified troponin I (TnI) and troponin T (TnT) in the serum. The National Academy of Clinical Biochemistry (NABC) working with ESC and American Heart Association (AHA) has recommended adoption of 99th percentile upper reference limit as the recommended cut off for a positive troponin result, based on findings from the GUSTO IV and TACTICS-TIMI 18 trial. An elevated troponin at baseline is an independent predictor of mortality even in patients with chest pain and STEMI who were eligible for reperfusion therapy [44,45]. The GUSTO 11A, GUSTO IV ACS and FRISC trial all demonstrated a direct correlation between the level of TnI or TnT and the incidence of cardiac morbidity and death post ACS [45]. In essence troponin rise post revascularization has been associated with IS and LVEF and therefore has a role in risk stratification and prognosis.

#### Other plasma biomarkers

There are several other plasma biomarkers including C-reactive protein (CRP), N-terminal pro-brain natriuretic peptide (NT-proBNP), cardiac troponin T and I, glucose which may have a role for risk stratification in STEMI. However, most of these markers are limited since there is a delay in enzymatic rise. For example, an elevated troponin can occur as early as 2–3 h versus 6–9 h in creatine kinase-MB. As such, most institutions have relied solely on troponins and discontinued the use of other cardiac markers. Nevertheless, creatine kinase-MB assessment at a single time point has been shown to be a good predictor of IS at 6 months on STEMI patients treated with PCI [46].

NT-proBNP concentration correlates with acute and chronic IS and myocardial function after AMI. Moreover, NT-proBNP concentrations are inversely associated with the potential for recovery of LV systolic function post AMI [47].

CRP is a nonspecific marker of inflammation, which has also been investigated as a potential marker in STEMI. Studies have illustrated that CRP predicted adverse cardiac events, acute MI and HF [48,49]. However, since it is a nonspecific marker it limits the use on the diagnosis of acute MI, unless used in conjunction with troponin or BNP.

#### Novel cytokines

Myeloperoxidase (MPO) is a leukocyte enzyme and has been subject to investigation as a novel cardiac marker. This enzyme generates reactant oxidant species and has also been linked to plaque instability as well as vasoconstriction from nitric oxide depletion. Elevated MPO has been shown to independently predict increased risk for major cardiac events, including MI, reinfarction and need for revascularization [50]. In fact, plasma MPO levels independently predict in-hospital mortality in STEMI patients treated by primary PCI [51].

Galectin-3 plays a role in the intracellular pathways of LV remodeling. This is also an emerging biomarker strongly associated with mortality already approved in the risk stratification of HF [52]. It has been shown to be a strong and independent predictor for LVEF after 4 months in patients with a first MI. When measured following STEMI, a high plasma galectin-3 predicts greater 30 days morbidity and mortality and increased HF incidence at a median of 2 years [53].

## Scoring systems

### GRACE score

There are several scoring systems in STEMI, allowing clinicians to estimate individual probability of unwanted outcomes. In ACS patients, the GRACE (or GRACE 2.0 score) can predict the outcomes of death, or MI over an extended 1 year and also death over a 3-year period from hospital admission. The clinical variables include age, heart rate, systolic blood pressure, creatinine, congestive HF, cardiac arrest, ST-segment deviation and elevated enzyme/cardiac biomarkers. The use of the GRACE score is recommended in the ESC guidelines, AHA and American College of Cardiology guidelines [1]. The GRACE risk score provides the most accurate stratification of risk both on admission and at discharge due to its good discriminative power [54]. The addition of biomarkers (e.g., NT-proBNP) has been shown to further enhance the discriminative power of the GRACE score and improve long-term risk prediction [55]. Further risk scoring methods are outlined in Table 2.

**Table 2.** **Risk scoring methods.**

**Risk scoring methods**	**Definition**
TIMI risk score	An additive scoring system using only six variables. It excludes Killip class, heart rate and systolic blood pressure as risk factors, leading to an inferior discriminative accuracy as compared with the GRACE risk score. However, it is simple to use and strongly correlates with mortality at 30 days

CADILLAC score	Predicts 30-day and 1-year mortality after primary PCI using the following identified variables: age >65 years (2 points), Killip class 2/3 (3 points), baseline LV ejection fraction (during left ventriculography) <40% (4 points), anemia (2 points), renal insufficiency (3 points), triple vessel disease (2 points) and post procedural TIMI flow grade (2 points). This criterion excludes high-risk patients with recent stroke, known renal dysfunction, cardiogenic shock, complex coronary anatomy and those undergoing rescue PCI

PAMI risk score	Derived from amalgamated data from various PAMI trials to determine 6-month mortality following primary PCI. It comprises of the following: age >75 years (7 points), age 65–75 years (3 points), Killip class >1 (2 points), heart rate >100 bpm (2 points), DM (2 points), anterior MI or LBBB (2 points)

SYNTAX score	An anatomical model designed to decide on the optimal mode of revascularization in complex coronary artery disease, e.g., three-vessel disease or left main stem disease. Despite having a role in risk stratifying STEMI patients with PCI, it can be further improved through a combination of clinical variables

GUSTO-1	Derived from the GUSTO trial, aims to predict 1-year survival in STEMI patients. Predictors include older age lower weight, Killip class, admission lower blood pressure, higher heart rate, QRS duration, smoking, history of hypertension or cerebrovascular disease or arrhythmia

DM: Diabetes mellitus; GRACE: Global Registry of Acute Coronary Event; LBBB: Left bundle branch block; LV: Left ventricle; MI: Myocardial infarction; PCI: Percutaneous coronary intervention; RBBB: Right bundle branch block; STEMI: ST-elevation myocardial infarction; TIMI: Thrombolysis in myocardial infarction.

## Predischarge

### Echocardiography

Echocardiography, a noninvasive tool, remains the main investigation choice in diagnosing complications of MI and evaluating cardiac function MI. Evidence has shown that the use of echocardiography predischarge is valuable in risk stratification post STEMI [56].

#### Left ventricular ejection fraction

LVEF measured by echocardiography predischarge is a valid indicator of IS and has shown by multiple studies to be an established predictor of cardiac remodeling and adverse clinical outcome. A prospective cohort study by Vakili *et al.* which included 304 STEMI patients reperfused by primary PCI, showed that LVEF ≤50% was associated with high rates of in-hospital adverse events including death [57]. LVEF has also been postulated to be a predictor of adverse cardiac remodeling. A study by Wita *et al.*, has shown that a lower LVEF on discharge in STEMI patients was independently associated with LV remodeling at 6 months [58].

#### LV global longitudinal strain

The use of novel imaging techniques, such as speckle-tracking echocardiography, allows the measurement of LV global longitudinal strain (LVGLS), which is an accurate index of global LV function [59]. This has been shown to be a potential predictor of LV remodeling in STEMI patients. In a cohort of 1041 primary PCI-treated STEMI patients, Joyce *et al.* showed that LVGLS predischarge was independently associated with LV dilatation at follow-up as well as a potential marker of IS [60]. Furthermore, a study by Munk *et al.*, (n = 227) showed that LVGLS measured at day 1 in STEMI patients was superior to LVEF in predicting 30-day IS [61]. Since LVGLS provides a semi-automated, quantitative measure of LV function without the use of ionizing radiation, contrast media or pharmacological stressors, it has proven to be a valuable tool for early risk evaluation in STEMI patients.

#### LV volume

Post infarct remodeling is a complex dynamic process which involves global LV enlargement post MI. It has been shown by McKay *et al.*, to be associated with IS since myocardium loss increases LV loading, which stimulates compensatory changes that may cause HF [62]. A study by Brzezinska *et al.*, (n = 132) has showed that an increase in LV end-diastolic volume index ≥80 ml/m^2^ at discharge was an independent predictor of progressive LV dilatation in STEMI patients [63]. However, this is still controversial, since Bolognese *et al.* have identified that LV end-diastolic volume index ≥80 ml/m^2^ predischarge was not a significant predictor of progressive LV dilatation [64].

#### Complications of MI: LVT, LV aneurysm, papillary muscle rupture & VSD

Echocardiography is a readily available tool for the immediate diagnosis of STEMI complications. The diagnosis of left ventricular thrombus (LVT) has dramatically improved with the use of echo-contrast since it can detect LVT that is inconclusive on conventional echocardiography. Gianstefani *et al.* has shown the possibility of early detection of LVT predischarge in STEMI patients [65]. However, it did not demonstrate an increased mortality risk in the presence of LVT. This result was supported by a retrospective study by Garber *et al.*, which showed that LVT was not significantly associated with increased mortality in STEMI patients [66]. Despite this, early diagnosis of LVT by echocardiography is important for early treatment and risk stratification, since LVT is associated with increased morbidity [67].

Another complication of MI is the formation of LV aneurysm. True aneurysms usually involve the entire wall thickness and are described as dyskinetic areas of thinned myocardium, while pseudoaneurysms that are contained by overlying pericardium, usually a result of ventricular wall rupture and are prone to spontaneous rupture. Although there is not much literature analyzing STEMI patients with post MI LV aneurysm, Visser *et al.*, found that early aneurysm formation post MI is associated with high mortality in the short and long term [68].

### Cardiac MRI

The duration of a cardiac MRI scan is typically 30–60 min, therefore, only patients who are well enough to lie flat for this length of time may be considered. Echocardiography is the imaging modality of choice in the acutely unwell STEMI patient to assess for mechanical complications of MI. However, in stable patients, cardiac magnetic resonance (CMR) provides information on infarct pathology, as well as LV function and volume, which together enable a comprehensive assessment of the heart, and its’ potential for recovery, after acute STEMI. CMR is a comparatively expensive technology, often with limited availability in the clinic. As with any test, the use of CMR should be supported by evidence of benefit from stratified interventions, and in his regard, more research is warranted.

#### T2-weighted edema imaging for myocardial edema

T2-weighted (T2W) imaging depicts myocardial edema which results from tissue injury during ischemia and reperfusion. Studies into the prognostic value of T2W imaging post-STEMI are based on the concepts of area-at-risk (AAR) and MS. The myocardial AAR is defined as the entire area of hypoperfused myocardium during an MI. A landmark study by Aletras *et al.*, showed that, in canines subjected to coronary occlusion, the area of hyperintensity on T2W imaging was consistently larger than IS measured by late-gadolinium enhancement (LGE) and was consistent with histological measurement of the AAR [69]. The AAR could then be divided into areas of irreversible ischemia and reversible ischemia.

**Figure 2.**
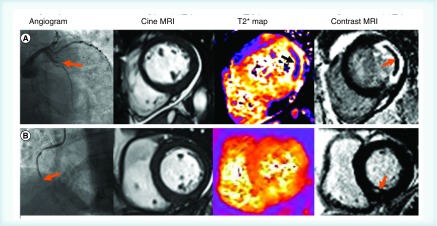
Two patients with acute ST-elevation myocardial infarction who had cardiac MRI 2 days post-event including T2* mapping. Angiography in patient A revealed an acutely occluded large obtuse marginal branch (orange arrow). Contrast MRI revealed a lateral infarct with significant microvascular obstruction (orange arrow). This corresponded to an area of intramyocardial hemorrhage as revealed by T2* mapping (black arrow). Patient B suffered an acute occlusion of the mid segment of the right coronary artery (orange arrow). Contrast MRI revealed a small infarct territory (orange arrow) with no intramyocardial hemorrhage visible on the T2* map.

Myocardial salvage, is defined as the difference between the final IS and the AAR, as demonstrated by a high T2W signal to indicate myocardial edema but no LGE signal to suggest irreversible myocardial injury (scar) (Figure 2). This concept is useful in the confirmation of recent ischemic injury, since it offers the ability to differentiate acute from chronic infarcts. Eitel *et al.*, found that in 208 acute STEMI survivors, the amount of MS was the strongest inverse predictor of major adverse cardiovascular events and mortality at 6-month follow-up, when included in a multivariable cox regression model with Killip class at admission, TFG after PCI, LVEF, ST-segment resolution and IS [70].

#### Late gadolinium enhancement for MVO & IS

LGE differentiates irreversibly damaged (nonviable) myocardium, from myocardium which is simply stunned following an acute ischemic insult, with acute reversible myocardial injury being T2W-edema positive but LGE-negative. LGE imaging also has the ability to identify microvascular obstruction (MVO). The presence of MVO represents more severe ischemic injury and is directly correlated with larger IS [71]. This can be recognized angiographically as the ‘no-reflow’ phenomenon, which is defined as a failure to perfuse at a microvascular level, despite a fully patent epicardial artery following revascularization. This is well established as a negative prognostic marker and is recognized as a central hypo-intense core (as shown by the dark zones) within bright regions of LGE [70]. Eitel *et al.* showed that in a large multicentre STEMI population (n = 738) reperfused by primary PCI, CMR markers of myocardial damage (IS and especially MVO) provide independent and incremental prognostic information in addition to clinical risk scores and LVEF [70]. Similarly, Van Kranenburg *et al.* showed that in 1025 reperfused STEMI patients, the presence of MVO was the strongest independent predictor of cardiac death and MACE at 2 years [72]. Novel quantitative T1 mapping techniques show promise for characterization of infarct pathology without the use of intravenous contrast agent. Carrick *et al.* showed that tissue pathology revealed by native T1 maps had similar prognostic value to MVO revealed by LGE CMR [73].

#### Myocardial hemorrhage with T2*-CMR

Myocardial hemorrhage is a complication of severe reperfusion injury in STEMI patients. The pathophysiology is closely intertwined with MVO; however, they are distinct entities, detected with different imaging sequences [74]. T2* CMR is the current gold standard for *in vivo* assessment of myocardial hemorrhage. Myocardial hemorrhage is associated with adverse remodeling and adverse health outcomes in the longer term [75]. Importantly, the occurrence of intramyocardial hemorrhage is prognostically more significant than MVO [76]. Indeed, the detection of intramyocardial hemorrhage and MVO may have a pivotal role in the risk assessment in STEMI survivors.

## Conclusion

Patients with acute STEMI are at risk of complications acutely and in the longer term. During emergency care, clinical judgement and immediately accessible tools, such as the ECG, chest x-ray and echocardiography, remain standard of care tests. In invasively managed patients, adjunctive functional tests, in other words, IMR, CFR, provide complementary information to the angiogram. Cardiac MRI is the reference test for the assessment of infarct pathology and viability. The use of any test for risk stratification should be linked with treatment, and more research is warranted to define evidence-based therapeutic interventions with stratified testing.

## Future perspective

STEMI is still one of the leading causes of morbidity and mortality worldwide. However, due to improved detection and timely reperfusion the mortality associated with STEMI is declining, although a substantial proportion of patients still develop chronic cardiac failure in the longer term. We forecast diagnostic accuracy will continue to evolve with the development of increasingly sensitive biomarkers of myocyte damage and LV dysfunction, ultimately allowing for a timelier diagnosis and prognostication. Coronary guidewire-based sensor technologies show promise as a new approach to risk assessment at the very earliest stage of acute MI management in the catheterization laboratory, immediately post reperfusion. Another development in the field will be the increasing use of noninvasive techniques for selected risk stratification in the early post-MI period, notably cardiac MRI. Cardiac MRI provides a wealth of information, including viability and potential for functional recovery, with a high degree of accuracy and also allowing repeat scanning since it uses nonionizing radiation. Cardiac MRI will play an evolving role in STEMI patients, especially through the continuing development of novel quantitative mapping techniques, allowing for a more objective assessment of myocardial injury and better tissue characterization.

**Executive summary**

**Background**

- In recent years the mortality associated with ST-elevation myocardial infarction (STEMI) is declining, in part through increasing symptom recognition, accurate diagnosis and effective timely reperfusion. Nevertheless, mortality still remains substantially high, especially in higher risk patients.

**Prehospital course**

- Initial presentation: early risk assessment at the time of first medical contact is vital and includes patient age, history of comorbidities as well as examination findings, including vital signs.

**Catheterization laboratory**

- Angiographic assessments of coronary flow and myocardial perfusion: thrombus in myocardial infarction (TIMI) flow is an angiographic measure of flow, with patients having higher TIMI flow grades linked to smaller infarct sizes (ISs), better ejection fractions (EFs) and shorter hospital stays. Myocardial blush grade is a measure of myocardial perfusion with score of 0 linked to higher mortality rates than compared with a myocardial blush grade of 1, 2 or 3.
- Coronary guidewire-based sensor technologies are emerging as new diagnostic tools for the invasive assessment of coronary artery disease in the setting of acute coronary syndromes. An elevated index of microcirculatory resistance at the time of primary percutaneous coronary intervention predicts poor long-term outcomes.

**Post reperfusion (coronary care unit)**

- Electrocardiogram monitoring should be continuous and acquired 60–90 min post reperfusion to asses for successful myocardial perfusion.
- Plasma biomarkers: cardiac troponin is the most commonly used plasma biomarker with an elevated troponin at baseline shown to be an independent predictor of mortality. There are several other plasma biomarkers including C-reactive protein, N-terminal pro-brain natriuretic peptide, cardiac troponin T and I, glucose which may also have a role for risk stratification in STEMI.
- Scoring systems: there are several scoring systems in STEMI, allowing clinicians to estimate individual probability of unwanted outcomes. The Global Registry of Acute Coronary Events risk score provides the most accurate stratification of risk both on admission and at discharge due to its good discriminative power.

**Pre discharge**

- Echocardiography: echocardiography is a noninvasive tool and remains the main investigation choice in diagnosing complications of myocardial infarction. Evidence has shown that the use of echocardiography predischarge is valuable in risk stratification post STEMI. For example, left ventricular EF measured by echocardiography predischarge is a valid indicator of IS and has shown by multiple studies to be an established predictor of cardiac remodeling and adverse clinical outcome.
- Cardiac MRI: cardiac MRI provides information on infarct pathology, as well as left ventricular function and volume, which together enables a comprehensive assessment of the heart, and its’ potential for recovery, after acute STEMI. Cardiac MRI can also detect infarct characteristics including myocardial hemorrhage, which is a complication of severe reperfusion injury in STEMI patients and is now emerging as an important prognostic marker.

**Conclusion**

- Patients with acute STEMI are at risk of complications acutely and in the longer term. During emergency care, clinical judgement and immediately accessible tools, such as the ECG, chest x-ray and echocardiography, remain standard of care tests. For invasively managed patients, adjunctive functional tests including index of microvascular resistance and coronary flow reserve can provide complementary information to the angiogram. However, the extent of their full role in the diagnosis and prognosis remains to be validated.
